# On the Potential of Surfers to Monitor Environmental Indicators in the Coastal Zone

**DOI:** 10.1371/journal.pone.0127706

**Published:** 2015-07-08

**Authors:** Robert J. W. Brewin, Lee de Mora, Thomas Jackson, Thomas G. Brewin, Jamie Shutler

**Affiliations:** 1 Plymouth Marine Laboratory, Plymouth, Devon, United Kingdom; 2 National Centre for Earth Observation, Plymouth Marine Laboratory, Plymouth, Devon, United Kingdom; 3 Chatham & Clarendon Grammar School, Ramsgate, Kent, United Kingdom; 4 College of Life and Environmental Sciences, University of Exeter, Cornwall Campus, Penryn, Cornwall, United Kingdom; Università di Genova, ITALY

## Abstract

The social and economic benefits of the coastal zone make it one of the most treasured environments on our planet. Yet it is vulnerable to increasing anthropogenic pressure and climate change. Coastal management aims to mitigate these pressures while augmenting the socio-economic benefits the coastal region has to offer. However, coastal management is challenged by inadequate sampling of key environmental indicators, partly due to issues relating to cost of data collection. Here, we investigate the use of recreational surfers as platforms to improve sampling coverage of environmental indicators in the coastal zone. We equipped a recreational surfer, based in the south west United Kingdom (UK), with a temperature sensor and Global Positioning System (GPS) device that they used when surfing for a period of one year (85 surfing sessions). The temperature sensor was used to derive estimates of sea-surface temperature (SST), an important environmental indicator, and the GPS device used to provide sample location and to extract information on surfer performance. SST data acquired by the surfer were compared with data from an oceanographic station in the south west UK and with satellite observations. Our results demonstrate: (i) high-quality SST data can be acquired by surfers using low cost sensors; and (ii) GPS data can provide information on surfing performance that may help motivate data collection by surfers. Using recent estimates of the UK surfing population, and frequency of surfer participation, we speculate around 40 million measurements on environmental indicators per year could be acquired at the UK coastline by surfers. This quantity of data is likely to enhance coastal monitoring and aid UK coastal management. Considering surfing is a world-wide sport, our results have global implications and the approach could be expanded to other popular marine recreational activities for coastal monitoring of environmental indicators.

## Introduction

The coastal zone is regarded as one of the most valuable and vulnerable habitats on Earth [1]. It contains the richest level of marine biodiversity [2, 3] and has a higher economic value per unit area than terrestrial and open-ocean ecosystems [4, 5]. The coastal zone supports a significant proportion of the world’s fish catch and is a source of non-renewable and renewable energy, waste disposal and recreation [6–10]. Human population densities in coastal regions (within 100 km distance of the coast and < 100 m above sea level) are estimated to be three times higher than global averages [11] and set to increase [12]. As a consequence the coastal zone is under increasing threat from: overfishing [6]; habitat degradation [13]; marine species loss [14, 15]; climate change [16]; harmful algal blooms [17]; hypoxia [18]; and eutrophication [19]. Coastal management is used to minimise the negative impacts of anthropogenic activity without compromising the socio-economic benefits of the coastal region [20].

Monitoring the coastal zone is fundamental to coastal management. Without adequate monitoring, environmental managers lack the information required to develop sufficient understanding for good management, or enable response to sudden (e.g. sporadic events) and long-term change (e.g. climate change). Environmental indicators are simple measures used to track the state of an environment [21]. In the coastal zone, these indicators can be physical (e.g. changes in land cover, currents, temperature, salinity, turbidity), biological (e.g. phytoplankton abundance and composition, macrophyte abundance) or chemical (e.g. nutrient concentrations, pH, toxic contaminants) [22]. There is high demand for observations on environmental indicators for coastal management of water quality, conservation, human resources and recreation (e.g. European Union Water Framework Directive) [22, 23]. Traditionally *in situ* measurements, acquired using conventional platforms such as research vessels or buoys, have been used to monitor environmental indicators. However, traditional methods for collecting *in situ* measurements are expensive and hampered by challenges in the coastal zone; for instance, from biofouling, and from the effects of tides, wave shoaling and coastal currents. The deployment and maintenance of such systems are also inherently expensive. Demand for observations on environmental indicators is not met by *in situ* datasets currently available [24]. Consequently, inadequate sampling coverage in the coastal zone is regarded as a major challenge facing coastal management [25].

To improve sampling coverage remote-sensing systems have been deployed from satellite, aircraft and at fixed positions along the coastline. Satellite remote-sensing of visible and thermal imagery is capable of providing affordable imagery with good temporal and geographic coverage, but is often limited by spatial resolution [26], and challenged by the optical complexity of coastal waters [27] and by atmospheric-correction [26, 28]. Aircraft-mounted sensors can significantly improve spatial coverage in coastal waters [29, 30], but are costly, especially when acquiring a high-temporal coverage. Fixed video systems are capable of improving spatial coverage [25, 31] and can extract information at low cost, on coastal morphology, currents and waves [32–34]. However, fixed video systems are limited in viewing range (∼ 2 km from the cameras in either direction [25]) and only available at specific locations. Furthermore, measurements of the ocean from remote-sensing platforms (satellite, aircraft and from fixed positions along the coastline) are limited to what can be measured using optical and infra-red radiation and require *in situ* data for calibration and validation. Other innovative solutions are needed to improve sampling coverage in the coastal zone, such as citizen science.

Citizen science is the outsourcing of a task once performed by a set of professionals to a large network of voluntary citizens. If carefully constructed, it can promote public understanding of science [35–37] and tackle costly, intractable and laborious research problems [38–40]. The generation of reliable scientific data through citizen science has contributed to unexpected insight and innovation, and high-quality research [41, 42]. Emerging technologies, such as mobile applications, wireless sensor networks, on-line computer/video gaming, and miniaturised environmental sensors, show great promise for advancing citizen science [43]. The influence of gaming and competition plays a large role in participant motivation [43–45], highlighting the benefits of incorporating recreation into citizen science [46].

In oceanography, citizen science has much untapped potential [47], especially when considering the high cost of oceanographic sampling (e.g. ship or boat hire) in comparison with many terrestrial-based sciences. In the UK alone, it has been estimated that 5.4 million people are involved in a recreational activity that requires direct interaction with the aquatic environment (both ocean and in-land waters), including some: ∼ 800,000 kayakers; ∼ 624,000 small-boat sailors; ∼ 518,000 surfers; ∼ 271,000 scuba divers; and 4.8 million outdoor swimmers [48]. Of all these major water-sport activities, surfing has the highest proportion of activity undertaken at the coastline [48]. The surfing community are also strong advocates of environmental monitoring (e.g. see Surfers Against Sewage [http://www.sas.org.uk/] and the Surfrider Foundation [http://www.surfrider.org/]), orchestrate their activities year round [49], and have an intrinsic interest in the functioning and state of the environment [50], making them a good target audience for citizen science projects in the coastal zone.

In this paper, we investigate the potential of using surfers as platforms to monitor environmental indicators in the coastal zone, with a view to enhance the sampling coverage required to improve coastal management. We focus our efforts on collection of sea-surface temperature (SST) data, considered an important environmental indicator for coastal management [22, 23], which plays a fundamental role in: the structuring of marine biodiversity in coastal environments [3]; the initiation and duration of the spring phytoplankton bloom [51]; the growth and metabolic rates of all trophic level species, from plankton [52, 53] to fish [54, 55]; the exchange of climatically important gases between the atmosphere and the ocean [56]; the local weather and climate [57]. Temperature is also a property that can be measured relatively easily (e.g. through measurements of electric resistance) and cheaply, making it ideal for citizen science-based projects. We equipped as surfer with a temperature sensor and Global Positioning System (GPS) device for a period of one year that they used when surfing. The SST data are compared with estimates from a local oceanographic station and satellite data, to determine if SST acquired from a surfer is reliable and what additional benefits it may bring. The GPS data is used to acquire information on surfer performance and used to illustrate potential motivation for data collection. Our results are extrapolated using estimates of the UK surfing population to demonstrate the potential of using surfers to improve sampling coverage of environmental indicators in the coastal zone. Finally, we discuss the implications of our results for other recreational water-sports and for monitoring environmental indicators not accessible by remote means.

## Materials and Methods

### Statistical tests

To compare SST data acquired by the surfer with those acquired from other sources, we used the squared Pearson correlation coefficient (*r* ^2^) and the Root Mean Square Error (Ψ), the latter calculated according to

$$\Psi ={\left[\frac{1}{N}\overset{N}{\underset{i=1}{\sum }}{\left(X_{i}^{E}-X_{i}^{M}\right)}^{2}\right]}^{1/2},$$ (1)

where, *X* is the variable (SST) and *N* is the number of samples. The superscripts *E* and *M* refer to two independent methods of measuring the same variable (e.g. one from the surfer and one from the satellite). The Ψ can also be partitioned into its precision and accuracy (or bias) components, such that Ψ^2^ = Δ^2^+*δ* ^2^, where the precision component Δ is expressed as

$$\Delta ={\left(\frac{1}{N}\sum_{i=1}^{N}{\left\{[X_{i}^{E}-(\frac{1}{N}\sum_{j=1}^{N}X_{j}^{E})]-[X_{i}^{M}-(\frac{1}{N}\sum_{k=1}^{N}X_{k}^{M})]\right\}}^{2}\right)}^{1/2},$$ (2)

and the accuracy (or bias) component as

$$\delta =\frac{1}{N}\sum_{i=1}^{N}(X_{i}^{E}-X_{i}^{M}).$$ (3)

Having a term available that distinguishes systematic (*δ*) and random (Δ) differences was found to be useful in the context of this study. We also computed the slope (*S*) and intercept (*I*) of a linear regression between *X* ^*E*^ and *X* ^*M*^. A slope (*S*) close to one and an intercept (*I*) close to zero is an indication that the two estimates of temperature agree well.

### Equipment

**Fig 1:**
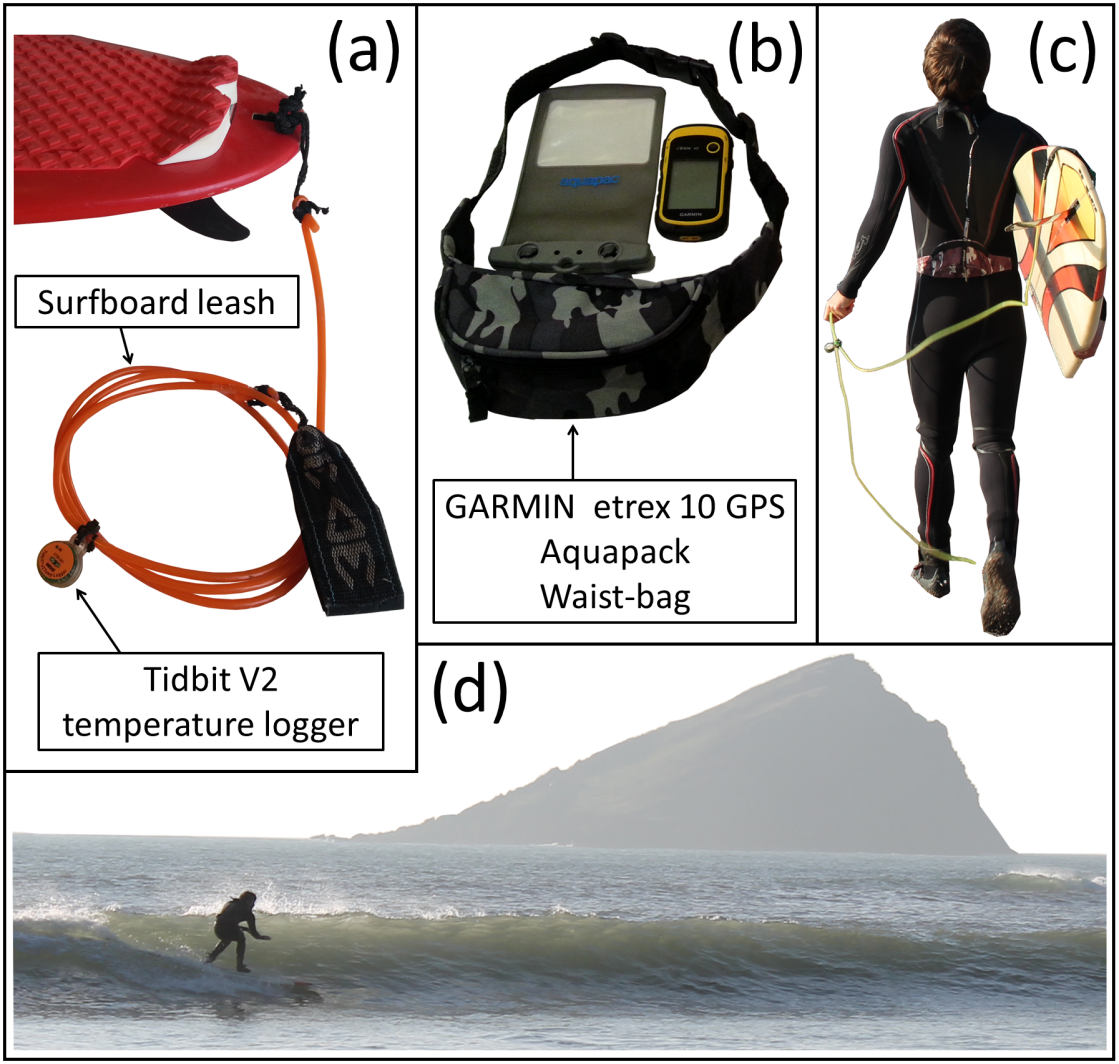
Equipment used in the study and surfer set-up. (a) Shows the Tidbit V2 temperature logger attached at mid-point to the surfboard leash. HOBOware software and HOBO USB Optic Base Station (BASE-U-4) were used by the surfer to launch the Tidbit V2 temperature logger prior to each session, and then to upload data post session. (b) Shows the GARMIN extrex 10 GPS, water-resistant Aquapac and waist-bag worn by the surfer. Information at one second intervals on location (latitude and longitude), time, distance, speed and orientation for each surf, were extracted from the GPS device post session. (c) Shows the surfer equipped with the sensors, and (d) shows the surfer collecting data during a session at Wembury beach. Consent to publication was obtained from the participant in this figure.

Following advice from the Faculty Research Ethics Committee for Health and Human Sciences Research at Plymouth University, UK, that ethical approval was not required for our study, a recreational surfer (lead author) was equipped with a UTBI-001 Tidbit V2 Temperature Data Logger and a Garmin etrex 10 GPS (Fig 1).

The Garmin etrex 10 device was used to extract GPS information. It contains an EGNOS-enabled GPS receiver, has HotFix satellite prediction and can track both GPS and GLONASS satellites simultaneously, allowing it to use 24 more satellites than using GPS alone. During each session, the Garmin etrex 10 device was stored in a water-resistant Aquapac inside a waist-bag worn by the surfer (Fig 1), and set to record GPS data at 1 second intervals. Information on location (latitude and longitude), time, distance, speed and orientation for each surf were extracted from the GPS device post session.

The Tidbit V2 temperature logger was attached, using cable-ties, at mid-point to the leash of the surfboard to ensure continuous contact with seawater when surfing (Fig 1), measuring temperature in the top metre of the water column. The waterproof Tidbit V2 temperature logger has an accuracy of 0.2°C over a range of 0–50°C, a resolution of ∼ 0.02°C at 25°C, a stability of ∼ 0.1°C per year, a response time of 5 minutes in water, and a battery life of ∼ 5 years at a > 1 minute logging interval. Three times during the period of study (May and August 2014 and January 2015), the Tidbit V2 temperature logger was compared with a VWR1620-200 traceable digital thermometer (NIST/ISO calibrated, with an accuracy of 0.05°C at the range of 0 to 100°C and a resolution of 0.001°C) at 1°C intervals from 6 to 25°C using a PolyScience temperature bath. On all three occasions, and over the 6 to 25°C temperature range, the systematic bias (*δ*) between the Tidbit V2 temperature logger and the VWR1620-200 traceable thermometer was < 0.05°C, lower than the accuracy of the VWR1620-200 traceable thermometer, with a precision (Δ) < 0.025°C and an error (Ψ) < 0.05°C. The slope (*S*) ranged from 0.997–1.000 and the intercept (*I*) 0.047–0.084°C for the three tests. Results from the comparison indicate the Tidbit V2 temperature logger performed with high accuracy, with low bias and that its performance was stable over the study period. HOBOware software and HOBO USB Optic Base Station (BASE-U-4) were used by the surfer to launch the Tidbit V2 temperature logger prior to each session, and then to upload data post session. Temperature data were collected at 10 second intervals during each surf.

### Study site

**Fig 2:**
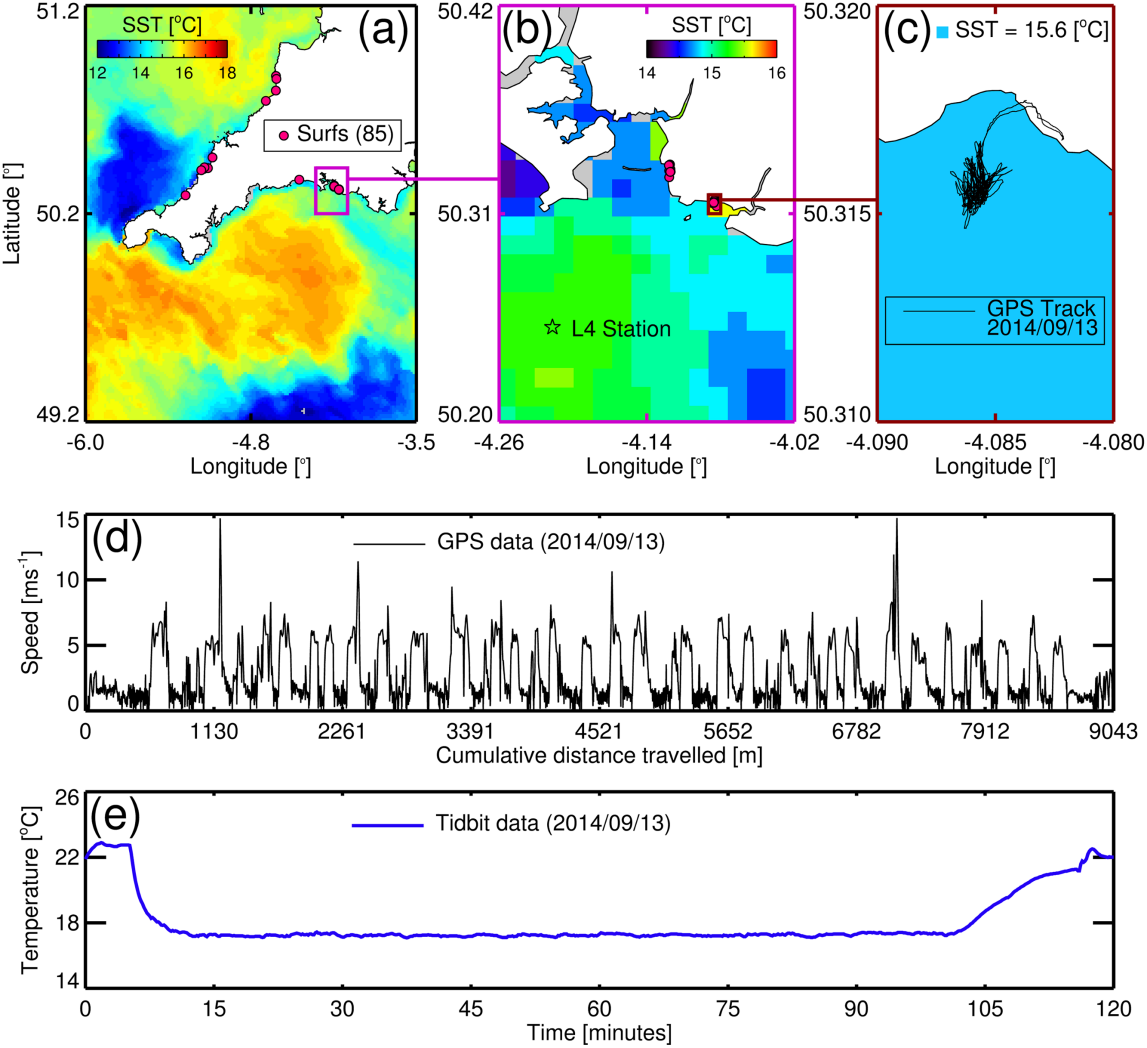
Study site and sampling locations with an example of GPS and temperature data collected by the surfer. (a) Shows the locations of the 85 surfing sessions in South West UK conducted during the study, overlain onto a NEODAAS AVHRR SST image taken on the 10^th^ September 2014. (b) Shows a plot of Plymouth and surrounding waters with locations of the surfing sessions near Plymouth and of station L4 in the Western Channel Observatory, with data from the AVHRR SST image (10^th^ September 2014). (c) Shows a plot of Wembury beach in Plymouth, with a GPS track taken by the surfer on the 13^th^ September 2014 overlain onto AVHRR SST estimate at Wembury beach (10^th^ September 2014). (d) Shows speed as a function of cumulative distance travelled for the GPS track taken on the 13^th^ September 2014, with the bumps in speed indicative of the surfer riding waves. (e) Shows a plot of temperature data collected by the surfer during the surf session on the 13^th^ September 2014.

The tagged surfer was stationed around the coastline of South West UK (Fig 2a). Between the 5th January 2014 and the 4th January 2015 the surfer orchestrated their recreational activity 85 times at a variety of locations (Fig 2a), with 74% of the surfs (63) conducted at Wembury beach near the city of Plymouth (Fig 2b and 2c) at a near weekly temporal sampling rate. A GPS track, taken on the 13th of September 2014, is shown in Fig 2c and illustrates how the surfer switched on the GPS device (and Tidbit V2 temperature logger) in the car park at Wembury (on land) then walked down to the beach and went surfing, before walking back to the car park and uploading the GPS and temperature data. Speed from the same GPS track is plotted as a function of cumulative distance travelled in Fig 2d, with the spikes in speed indicative of the surfer riding waves. The temperature data for the same session is also plotted as a function of time (Fig 2e) and illustrates a large change in temperature between switching the sensor on at the beginning of each session and entering the water (and exiting the water prior to switching the sensor off) with relatively stable temperature readings during the period the surfer was immersed in seawater.

### Temperature data processing

**Fig 3:**
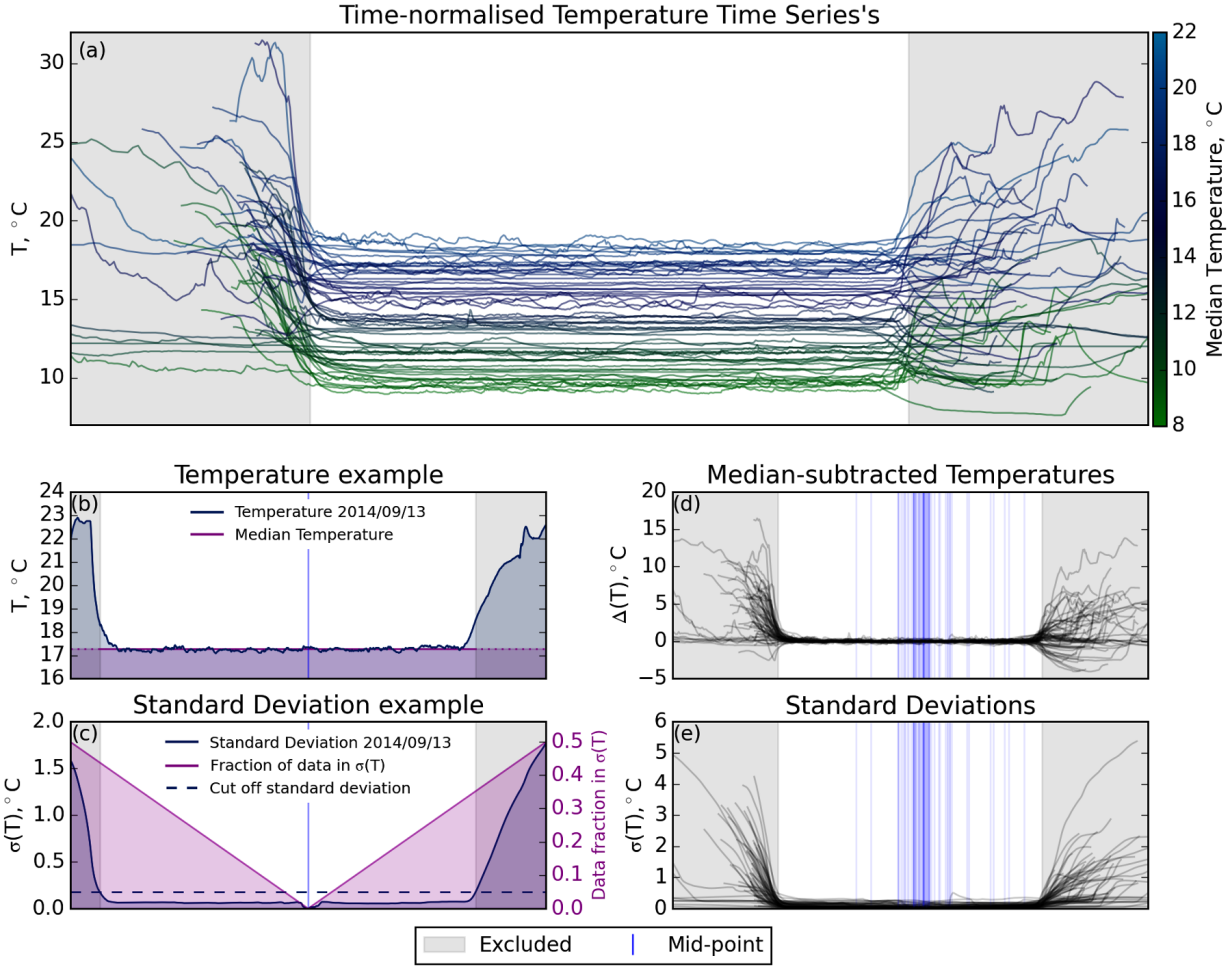
Processing method for the temperature data (denoted in the figure as T) acquired by the surfer. Figures (a), (d) and (e) were normalised such that the start (surfer entered the water and sensor equalised to water temperature) and end (surfer exited the water and sensor beginning to respond to air temperature) is at the same point on the graph (x-axis) for each session. (a) A superposition of all the temperature data acquired by the surfer during the study period at Wembury beach. (b) A typical temperature data set, acquired on the 13^th^ September 2014, showing the start and stop time of the surf (vertical grey lines), mid-point of data collection (blue line), excluded data (grey shaded areas) and the median of the data collected in the sea (considered as the SST). (c) Standard deviations computed using the processing method for data collected on 13^th^ September 2014, with the corresponding fraction of data used to calculate the standard deviations and the 10% threshold used to exclude data collected when the surfer was on land. (d) Shows the temperature of each session presented as in Fig 3a, but with the computed SST (median of remaining data) subtracted. (e) A superposition of the standard deviations for the data collected at Wembury beach.

Considering the Tidbit V2 temperature sensor was activated before entering the water, and deactivated after leaving the water, many of the initial and final temperature data were recorded when on land. Furthermore, as the sensor has a response time of up to 5 minutes in sea-water, the time between activation and sea-water equalisation varied among sessions. Therefore, the temperature data required processing to extract SST. While it is possible to use GPS data, together with tidal and shoreline information, to determine when the surfer was in the sea, we developed a GPS-independent SST extraction methodology. This was thought useful for occasions when either the clocks of the GPS and the temperature sensors disagree, or if temperatures were recorded without associated GPS. Fig 3a shows a superposition of all temperature data acquired by the surfer at Wembury beach during the study period. The data were normalised such that the start (surfer entered the water and sensor equalised to water temperature) and end (surfer exited the water and sensor beginning to respond to air temperature) is at the same point on the x-axis for each session. The colour scale of Fig 3a indicates the median of the remaining data (used to compute SST) after the exclusion of erroneous data. The methodology used to determine the start and stop times is described below.

Firstly, we make the assumption that the midpoint of the temperature data for each session occurred while the sensor was in the water. This was visually checked using GPS data and found to occur for every session at Wembury beach. Fig 3b shows an example of temperature data collected on the 13^th^ September with the midpoint shown as a vertical blue line. The temperature data for each session was then divided into two equal halves around the midpoint. For the initial half, every data point was removed sequentially and the standard deviation was calculated incrementally with the last data point representing the standard deviation of the midpoint (zero). For the second half, this procedure was repeated but in reverse. This method produced a list of standard deviations which are plotted (dark blue line) in Fig 3c for data collected on the 13^th^ September, with the fraction of data used to compute each standard deviation plotted as the purple line in Fig 3c. The period for which the surfer measured SST (immersed in sea-water) was then taken to be between the first and last points where the standard deviation was less than 10% of the largest standard deviation (Fig 3c dashed blue line). The cutoff of 10% was chosen based on a visual comparison with the timing of the first and last waves caught by the surfer, as estimated from the GPS data. All temperature measurements before and after these points were excluded (shown in the grey areas of Fig 3a–3e), and the median of the remaining data for each session used to compute SST.

Fig 3d shows the temperature of each session presented as in Fig 3a, but with the computed SST (median of remaining data) subtracted, and Fig 3e shows the standard deviations for the data collected at Wembury beach. These figures demonstrate that the method removes nearly all the erroneous initial and final points. Computing the median of the remaining data as the SST (rather than the mean of the remaining data) also minimises the influence of any erroneous data points that may pass through the processing at the margins of the dataset. The method was checked by visually inspecting the temperature data for each session with the GPS data. During one session, where air temperature and sea temperature were very similar (little variance), the method excluded a significant portion of the data collected in the sea. However, even in this singular case the computed SST using our method was not significantly different (within the accuracy of the sensor) from computing the median of the temperature data using the start (surfer entering the water) and end (surfer exiting the water) derived from the GPS (first and last wave caught).

Our method (Fig 3) is based on the assumptions that: (i) the temperature of the sensor in the sea is relatively stable compared with the variability caused by the transition from air to sea; (ii) that the mid-point of the dataset occurred in the sea; and (iii) duration in the sea is longer than duration out of the water. The method would need to be reviewed for conditions where these assumptions are breached.

### GPS data processing

The GPS data processing builds upon the recent work of Barlow *et al.*[58] to provide performance statistics on surf sessions. The GPS data processing was designed to account for variations in the range of surfing speeds due to differing surfer ability, surfboard types and ocean conditions. During each surf session GPS data were logged at a temporal resolution of one second. Each measurement provided a location, speed and bearing. Initially, a pre-processing filter was applied to remove any anomalous data with speeds greater than 15.3 m s^−1^, the speed of a swell with a significant wave height of 5 m [59], thought not to have occured during sampling.

**Fig 4:**
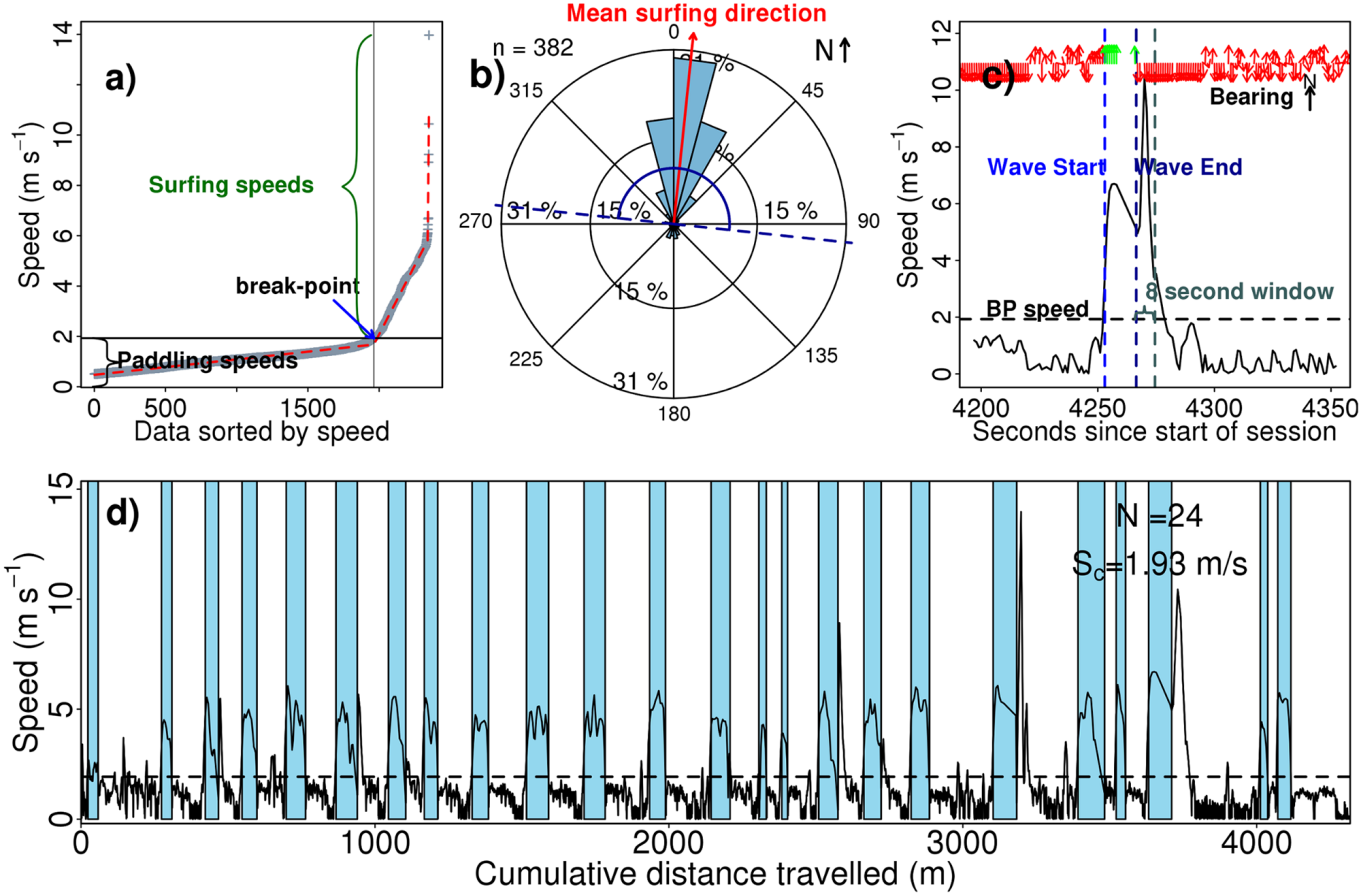
GPS processing used to estimate surfer performance statistics. An example from the 30^th^ August 2014. (a) Shows speed from the GPS plotted as a function of data sorted according to speed (grey crosses). Hinge functions were fitted to the data to partition it into three linear fits (red dashed lines), and identify two break-points. The first break-point (black solid line) in the hinge function was used to define the transition from paddling into surfing (wave riding). (b) Bearing (or direction) data for all surfing data points (where speed is greater than at the break-point in (a)). Red line indicates the average surfing direction during the session and the dark blue lines indicate the region with an angular window of ±90° either side of the mean surfing direction, defined as the permissible surfing directions and used to help control quality of the data. The letter n refers to number of data points measured while travelling above the break-point speed. (c) An example of a categorised wave during the session with speed (and bearing) plotted as a function of time. As the speed increases above the break-point speed (denoted BP) the wave starts (blue dashed line). In this case the wave is classified as ended (dark blue line) when the bearing falls outside the permissible surfing directions (see (b)), with the 8 second window (used to check (in this case) if bearing fell back inside the permissible surfing directions up to 8 seconds after it first fell outside) shown as the grey dashed line. (d) Shows speed as a function of cumulative distance travelled for the GPS track over the entire session, with waves classified in light blue shading. N refers to number of waves during the session and S_c_ (and the horizontal dashed line) denotes the break-point speed (see (a)) used to define the transition from paddling into surfing during this session.

Following the high speed filter, the speed at which a surfer could be classed as surfing (wave riding) was determined for each surf. This was done using all data where the surfer was not waiting (speeds greater than 0.5 m s^−1^[58]). The non-waiting speeds were arranged in ascending order and we implemented a version of the Jerome Friedman’s multivariate adaptive regression splines, to fit a number of hinge functions to the data (Fig 4a). The maximum number of model terms were set to five, to force the model to break the data into three regions. This allowed the identification of two break-points in the range of observed speeds. The first break-point represents the transition from paddling to surfing. Note that this transition is likely to change depending on wave size, surfboard type and surfer performance. We took the first break-point in the hinge function to define the minimum surfing speed for a given surf session. This was conducted independently for each surf session.

In addition to travelling at a required velocity, a surfer should also be travelling in the correct direction (approximately toward shore-line). Analysis of a number of individual sessions revealed occasional anomalous GPS data with speeds well above paddling velocities, headed away from shore, usually following the end of wave. As most of the data with surfing speeds are towards the beach, the bearings for all the data with speeds greater than the minimum surfing speed were averaged to give a mean surfing direction (Fig 4b). An angular window of ±90° either side of the mean surfing direction was defined as permissible surfing directions to allow for left and right rides, and prevent these anomalous GPS data for interfering with the performance statistics.

Following the establishment of these two criteria, the GPS data was analysed in chronological order. Once the minimum surfing speed had been crossed and the surfer was travelling in the right direction they were considered to be riding a wave. As the speed of a surfer may vary during a single wave, with some manoeuvres requiring the surfer to slow (or stall) the board, or to change direction sharply (possibly outside of the permissible surfing directions), the surfer speed was allowed to drop below the threshold velocity and outside permitted direction for up to eight seconds before a wave was considered finished (Fig 4c). Note that this does not result in an eight second addition to the duration of each wave, as each wave was terminated at the last valid point.

Following wave identification, any ride lasting less than four seconds [58] was counted as a failed wave and not included in the riding statistics. Having determined the beginning and end of each wave, the wave statistics and the total session information were computed. An example of performance statistics for a full surfing session is shown in Fig 4d. For the calculation of whole sessions statistics, the start of the session was set at two minutes before the first wave and the end at two minutes after the last wave, acknowledging that there is likely to be variability in this assumption. From the GPS processing a summary was produced for each surf session and the results from these were combined to produce an annual summary (number of sessions, waves caught, total ride time and distance). To put the performance statistics of the surfer derived using the GPS in the context of the wider surfing community, the tagged surfer was rated according to the Hutt *et al.*[60] surfer skill rating scale, that varies from 1–10. A rating of between 5 and 6 (intermediate level) was assigned to the surfer.

### Additional data sources

For comparison with the temperature data measured by the surfer at Wembury beach, SST data were acquired from two other sources: station L4 in the Western Channel Observatory (WCO) and from satellite observations of thermal infra-red radiation. Station L4 is a coastal station located ∼ 12 km south west of Wembury beach (Fig 2b) and forms part of the WCO, an oceanographic time series and marine biodiversity reference site in the Western English Channel [61, 62]. An autonomous buoy is operated at station L4 equipped with a WET Labs Water Quality Monitor (WQM), which incorporates WET Labs’ fluorometer-turbidity and Sea-Bird’s CTD sensors, providing temperature, salinity, depth, dissolved oxygen, chlorophyll fluorescence, turbidity and backscattering data. The WQM records SST at hourly intervals, with an accuracy of 0.002°C at a range of -5 to 35°C, and a resolution of 0.001°C. The buoy at station L4 was brought to shore in November 2013 for maintenance and was ready for redeployed in December 2013. However, due to the large storm events of winter 2013–2014, the buoy was not redeployed until March 2014. SST data were acquired for the period 11^th^ March 2014 to 4^th^ January 2015. Daily median SST were extracted from the time series, and we also extracted SST data at the hour closest in time to that acquired by the surfer at Wembury beach during the study period.

Advanced Very High Resolution Radiometer (AVHRR), daily, ∼ 1 km mapped, SST data were acquired from the NERC Earth Observation Data Acquisition and Analysis Service (NEODAAS [63]). This data were received at Dundee and processed in near-real time at Plymouth Marine Laboratory. An example of an AVHRR image, processed by NEODAAS over the South West UK for 10^th^ of September 2014, is provided in Fig 2a–2c. A daily time series of AVHRR SST data were extracted for station L4 and Wembury beach. This time series represented an average of a box of pixels (3 × 3) centred at station L4 (latitude = 50.25, longitude = -4.2167) and Wembury beach (latitude = 50.3160, longitude = -4.0854). Following standard methods, we used a multi-pixel box to increase the possibility of an *in situ* measurement (taken either at station L4 or Wembury) being available for comparison with the AVHRR data [64].

## Results and Discussion

### Sea Surface Temperature (SST) results

**Fig 5:**
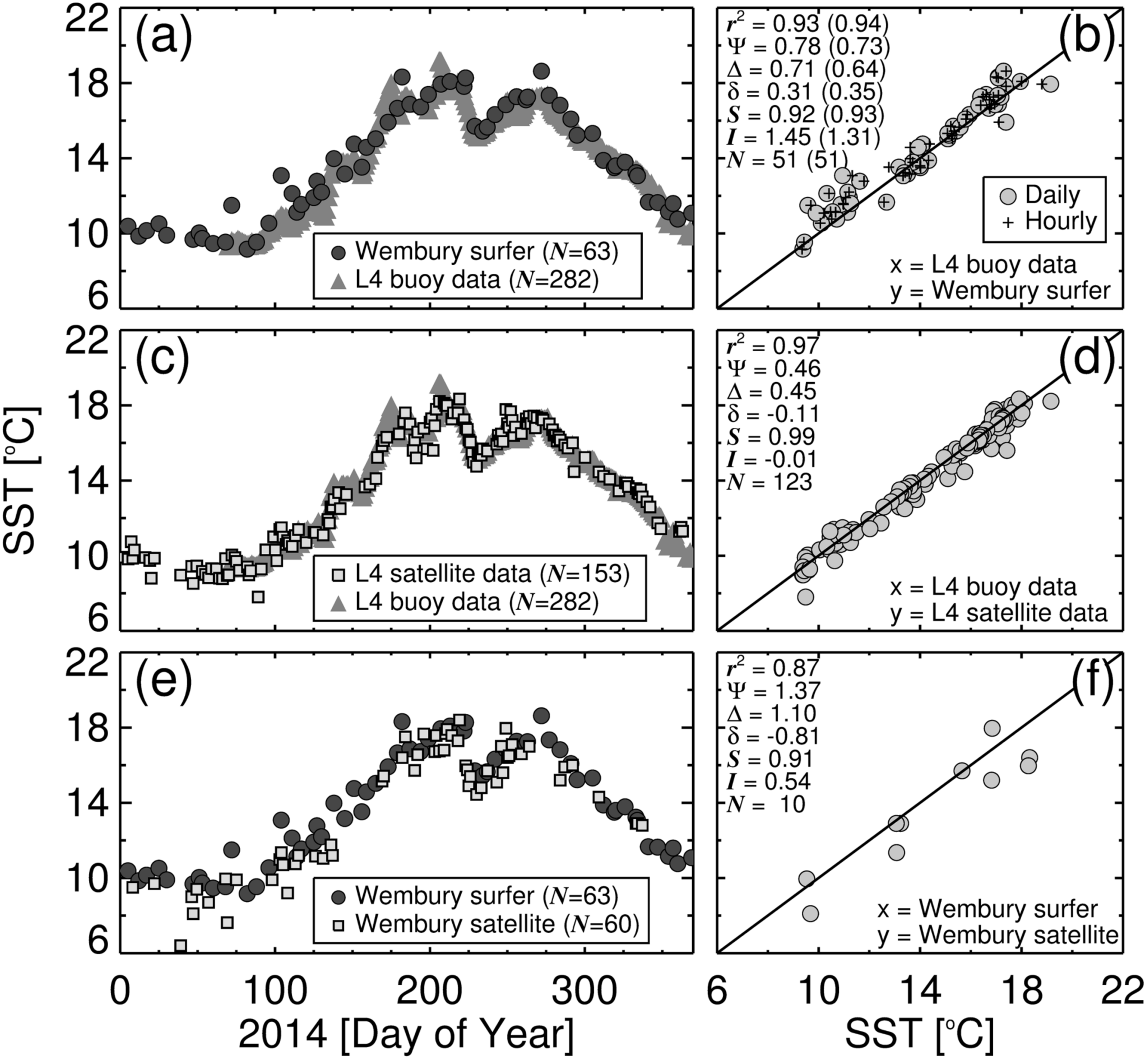
Sea surface temperature (SST) results from the study. (a) Shows the time series of SST acquired by the surfer at Wembury beach overlain onto the daily median SST data from station L4 during the study period (*N* refers to the number of samples). (b) Shows a scatter plot of daily match-ups between SST acquired by the surfer at Wembury beach and SST data from station L4. Bracketed statistics refer to use of hourly match-ups between the two datasets. (c) Shows the time series of SST from satellite (AVHRR) at station L4 overlain onto the daily median SST data from station L4 (buoy) during the study period. (d) Shows a scatter plot of daily match-ups between SST from satellite (AVHRR) at station L4 and SST data from the buoy at station L4. (e) Shows the time series of SST from satellite (AVHRR) at Wembury beach overlain onto SST acquired by the surfer at Wembury beach during the study period. (f) Shows a scatter plot of daily match-ups between SST from satellite (AVHRR) at Wembury beach and SST data acquired by the surfer at Wembury beach. Statistics are denoted as follows: *r* ^2^ is the squared Pearson correlation coefficient; Ψ is the Root Mean Square Error; Δ is the unbiased Root Mean Square Error; *δ* is the bias; *S* and *I* are the slope and intercept of a linear regression respectively; and *N* refers to the number of match-ups.

SST acquired by the surfer at Wembury beach over the study period is plotted in Fig 5a. In January 2014 (day 5) the SST was around 10°C and dropped to approximately 9.5°C by early March (day 60). Between March and July (day 60–210), the SST increased steadily from 9.5°C to 18°C, with a few sporadic increases observed over the period (Fig 5a). In early August (day 225) SST dropped rapidly from 18°C to 15°C, then rose back to approximately 18°C by late September (day 272). Between October 2014 and January 2015 (day 277 to > 365) there was a steady decline in SST from around 18°C to 11°C.

The time series of SST acquired by the surfer at Wembury beach is overlain onto the daily median SST data from station L4 (Fig 5a). There is good agreement between the two SST estimates, as illustrated by scatter plots between daily match-ups (Fig 5b). Over the seasonal cycle the SST data collected by the surfer at Wembury beach explained 93% of the variance in the SST data at station L4, with an error (Ψ) of 0.78°C and a bias (*δ*) of 0.31°C. When extracting station L4 SST data at the corresponding hour the surfer was immersed in the water (as opposed to comparing daily median estimates from station L4), statistical results improved further, with the surfer explaining 94% of the variance in the SST data at station L4, with an error (Ψ) of 0.73°C (Fig 5b). The SST data collected by the surfer at Wembury beach is seen to capture the general seasonal cycle (lower SST in the winter, higher SST in the summer) and also abrupt changes, such as the decrease in SST in August (day 225 Fig 5a) seen in the station L4 data. Good agreement between the two datasets illustrate the potential of using surfers to acquire high-quality SST data in the coastal environment.

Whereas the statistical tests confirm that SST from station L4 data agreed well with the SST data collected by the surfer at Wembury beach, there were still some marked differences (Fig 5a). During spring, a few sporadic increases in SST of around 2°C were observed in the Wembury time series, for instance, day 72 and 104 (see Fig 5a). Both these measurements coincide with small wave heights and clear skies (data not shown), with a late morning low tide and the surfing session occurring during mid-high tide in either the afternoon or evening. In both cases, it may be that the exposed inter-tidal land (which has a much lower heat capacity than the ocean) was warmed prior to the rising afternoon tide during which the surf took place, which together with a diurnal increase in water column temperature, possibly resulted in a localised increase in SST during the later period of the day. Sporadic increases in SST close to the coastline are not surprising when considering the multiple factors that may influence coastal temperature (e.g. land-sea temperature exchange and localised run-off from rainfall) that may not be captured by *in situ* instruments situated further offshore (e.g. at station L4). In some regions, data on differences between coastal and offshore SST may provide useful information regarding freshwater discharge (e.g. close to estuaries), variations in coastal currents, and the location and intensity of tidal mixing fronts [62, 65, 66]. All of which have implications for biological productivity and the transportation of pollutants and contaminants. Monitoring SST in coastal regions, relative to offshore regions, may also benefit marine recreation (e.g. useful information for water bathers).

The time series of satellite-derived SST at station L4 is overlain onto the daily median *in situ* SST data from the station L4 buoy in Fig 5c, and a scatter plot of the two SST estimates is shown in Fig 5d. At station L4, the satellite data is in very good agreement with the *in situ* observations, explaining 97% of the variance in the SST data with an error (Ψ) of 0.46°C, a bias (*δ*) close to zero, a slope (*S*) close to one, and an intercept (*I*) close to zero (Fig 5d). By contrast, comparisons of satellite SST and *in situ* SST (collected by the surfer) at Wembury beach are not so good (Fig 5e and 5f), with the satellite data explaining only 87% of the variance in the *in situ* SST with a higher error (Ψ = 1.37°C) and a large bias (*δ* = −0.81°C, Fig 5f). Furthermore, in comparison with 153 available satellite observations at station L4 over the study period, only 60 satellite observations were available at Wembury beach (Fig 5c and 5e). It is common to observe a fewer number of satellite observations in coastal regions as land-sea adjacency complicates the signal received by the satellite sensor and the nature of the aerosol composition at the coastline complicates atmospheric correction [26]. Higher errors (both systematic (*δ*) and random (Δ)) in satellite-derived SST at Wembury and fewer observations, when compared with those at station L4, further emphasize a need for *in situ* SST observations at coastal regions, which could be acquired by recreational waters-users such as surfers.

SST is one of the most important characteristics of an aquatic system. It is considered by the Global Climate Observing System as an essential climate variable [67], influencing: dissolved oxygen levels; the solubility and reaction rates of chemicals; the metabolism, growth and reproduction of marine organisms [52, 53]; and water density and stratification, which impact coastal physics and the transport of nutrients, contaminants and pollutants. Variations in nearshore SST have been correlated with coral bleaching events [68] and unusual, and sometimes harmful, algae blooms [69]. Monitoring SST in the nearshore region is of particular importance considering its high level of biological diversity, productivity, and economic value, when compared with open-ocean environments [2–5], and its vulnerability from a rising human population and climate change.

In addition to SST, it is feasible to monitor other environmental indicators by using recreational water-users, such as surfers. Salinity, a valuable environmental indicator in the coastal environment [70], can be estimated from measurements of conductivity. The acidity (pH) of seawater, which influences metabolic rates and immune responses of some organisms [71] and calcification [72], can also be measured electronically using a pH meter. In fact, as part of the Wendy Schimdt Ocean Health XPRIZE, there are ongoing efforts to develop a surfboard fin capable of simultaneously measuring temperature, salinity, and pH [73]. Monitoring nutrient concentrations and toxic pollutants is important for management of eutrophication and water quality. Recent advances in miniaturised technology [74, 75] may permit future measurements of nutrient concentrations and toxic pollutants from platforms such as surfboards. Large-scale data collection by recreational water-users on environmental indicators has the potential to improve our understanding of the coastal system, that is currently based on observations with suboptimal spatial and temporal coverage. Should a scientific question arise on a component of the coastal system, it may be possible to address this question by equipping recreational water-users with miniature sensors suitable for measuring the environmental indicator pertinent to the question.

#### GPS results

Annual surfer performance statistics derived from the GPS processing are provided in Table 1. Over the entire year (85 surf sessions), the surfer caught approximately 2012 waves, surfed for 90.6 hours (nearly 4 days), covered a distance of 375.8 km of which they rode 110.8 km. We conducted a sensitivity analysis of the fixed parameters involved in the GPS processing (see Table 1) and found the GPS processing method relatively robust, with minor effects on the annual statistics (Table 1).

**Table 1:** Annual surfer performance statistics.

**Surfer Performance variables**	**This Study^1^,^2^**
Total number of surfing sessions	85
Total number of waves caught	2012 ±73
Total time spent in the ocean (hours)	90.6 ±0.9
Total time spent riding waves (hours)	6.8 ±0.16
Total water distance covered (km)	375.8 ±2.4
Total riding distance covered (km)	110.8 ±2.1
Total paddling distance covered (km)	134.7 ±1.1
Total waiting distance covered (km)	81.1 ±1.2
Total miscellaneous distance covered (km)	49.1 ±1.8

^1^ Statistics based on one year of data collected by the same surfer of an intermediate level according to the Hutt *et al.* [60] surfer skill rating scale.

^2^ Uncertainty (±) was computed based on a sensitivity analysis of fixed parameters used in the GPS processing, with the following parameters varied: the minimum moving speed (fixed at 0.5 ms^−1^) varied between 0.2–1.0 ms^−1^, at 0.2 ms^−1^ intervals; the pre-processing high speed filter (fixed at 15.3 ms^−1^) varied between 10–18 ms^−1^, at 2 ms^−1^ intervals; the minimum duration of a classified wave (fixed at 4s) varied between 3–6 s, at 1s intervals; and the duration the surfer was allowed to fall below the threshold velocity and outside the permissible directions (fixed at 8s) varied between 6–10 s, at 1s intervals. For each sensitivity run, one parameter was varied while keeping others fixed at their default value. In total 17 sensitivity runs were conducted and for each of the surfer performance variables, standard deviations were computed based on the 17 runs and are provided as ± values in the table.

To assess the performance of our GPS method relative to those in the literature, we computed surfer performance metrics for comparison with Barlow *et al.*[58] (Table 2). In general, our results are remarkably similar with those of Barlow *et al.*[58] (see Table 2). Some level of similarity can be expected, when considering both methods rely on GPS data and there were similarities in data processing, yet the study of Barlow *et al.*[58] was based on 60 surfing sessions and 39 recreational surfers of varied ability, whereas our study was based on 85 surfing sessions by the same surfer, of an intermediate level [60]. Statistical results derived here (Table 2) are also comparable with those derived elsewhere [76, 77], especially when considering differences in surfer ability among studies.

**Table 2:** Surfer performance statistics derived in this study compared with those of Barlow *et al.* [58].

**Surfer Performance statistics**	**Barlow *et al.* [58]^1^**	**This Study^2^**
Number of rides (per hour)	20.6 ±11.41	21.8 ±5.6
Maximum of ride speed (m s^−1^)	6.1 ±1.2	7.7 ±2.3
Mean ride time (s)	13.0 ±5.0	11.7 ±2.7
Maximum ride time (s)	27.3 ±13.3	24.8 ±8.6
Mean ride distance (m)	54.8 ±25.4	51.9 ±14.6
Maximum ride distance (m)	117.7 ±63.4	105.8 ±39.1
Total distance covered whilst surfing (%)	25.6 ±9.6	27.8 ±8.1
Total time spent waiting (%)	41.8 ±9.8	59.3 ±9.7
Total time spent paddling (%)	47.0 ±6.1	29.4 ±7.5
Total time spent riding (%)	8.1 ±5.3	7.2 ±2.6
Total time miscellaneous (%)	3.1 ±1.9	4.1 ±3.6
Surfing cut-off speed^3^ (m s^−1^)	2.5	2.1 ±0.3

^1^ The study of Barlow *et al.* [58] was based on 60 surfing sessions and 39 recreational surfers of varied ability.

^2^ This study was based on 85 surfing sessions by the same surfer, of an intermediate level according to the Hutt *et al.* [60] surfer skill rating scale.

^3^ The study of Barlow *et al.* [58] used an absolute cut-off speed of 2.5 m s^−1^, whereas in this study the cut-off was determined independently for each session by use of hinge functions.

As highlighted in previous studies [58, 77], surfing statistics acquired from GPS data have use in monitoring fitness and performance. The prevalence of technology in recreation and leisure has proliferated in recent years with the development of mobile application technology. Mobile phone applications such as Strava, a GPS enabled smart-phone application designed to track recreational activities such as cycling and running, are becoming increasing popular. Coupled with developments in social media (e.g. Twitter and Facebook) and website visualisation (e.g. Google Earth), users of GPS enabled mobile applications can view and share their GPS track and performance. This has led to the use of mobile applications for recreational competition, for instance, the Strava software “King Of The Mountain” that is designed for users to compete with each other over who can cycle a particular route the quickest. The company RipCurl has introduced a GPS surf watch (http://www.ripcurl.com/searchgps-1.html) and other GPS-based devices are becoming available for monitoring surfer performance (e.g. the action sports tracker (TRACE) http://www.traceup.com/). It is likely similar applications will soon be available for surfers to compare their performance and compete. In citizen science, the influence of gaming and competition plays a large role in participant motivation [43–45]. Use of mobile GPS technology in surfing for competition could drastically enhance participation in a marine-based citizen science project focused on monitoring environmental indicators like SST.

### Potential of surfers to monitor environmental indicators

The equipment and approach used in our study (Fig 1) was designed to test the feasibility of using surfers to monitor environmental indicators in the coastal zone. It would likely require modification for widespread use. For instance, sensor launch, data offload and data post-processing (both SST and GPS) currently require a small investment of time before and after each surfing session, which may discourage citizen uptake. By leveraging emerging citizen-based technologies (e.g. mobile phone applications) and commercial GPS equipment, making use of established methods for data processing, sensor communication and data storage, this could be made quicker, easier and more efficient. As thermistor-based devices become cheaper and more widespread, equipment costs for measuring SST could be reduced further, but would need to balance against data quality and sensor durability. Considering the surfer as a platform, mounted environmental sensors need to be unobtrusive, so as not to interfere with surfer performance and discourage uptake. We found that the Tidbit V2 temperature logger (size = 30 × 41 × 17 mm, weight = 23 g) met this requirement, but not all sensors are likely to. Furthermore, any widespread data collection by surfers is likely to be biased toward conditions and locations preferable for surfing, and subsequent data analysis should consider associated biases. This issue could be minimised if the approach was expanded to other popular marine recreational activities, which take place in a variety of maritime conditions.

Results from this study demonstrate that it is feasible to use surfers as a platform to acquire high-quality data on environmental indicators in the coastal environment (Fig 5). If the approach was scaled-up to a large citizen-science based project, it would be pertinent to gauge the potential in data acquisition by surfers. Knowledge on the global surfing population and the frequency at which surfers partake in their activity is relatively scarce. However, in certain regions, such as in the UK, statistical estimates of surfers are available. For instance, it has been estimated that there are in the region of 500,000 to 700,000 surfers in the UK [48, 78].

**Fig 6:**
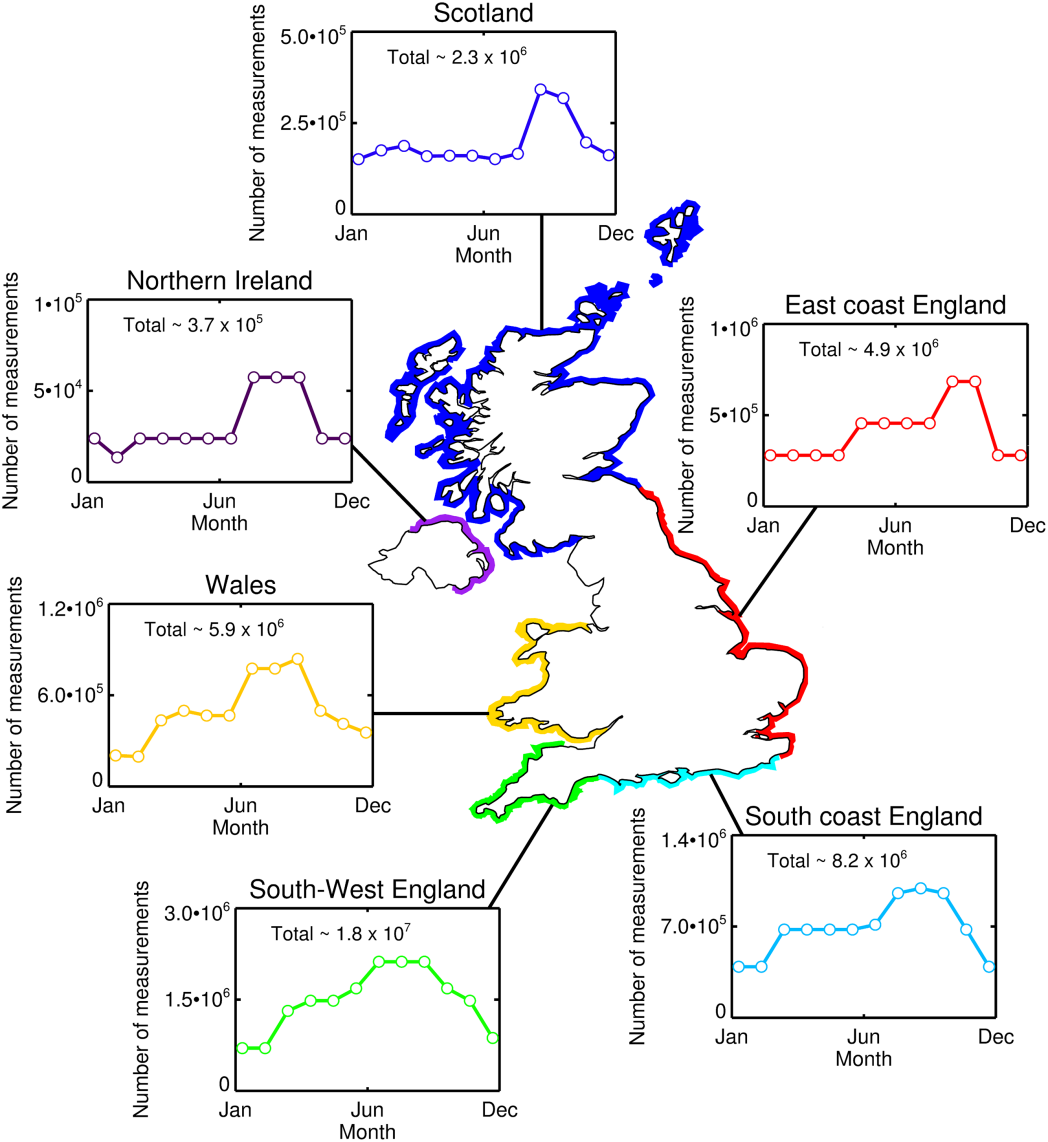
Estimates of the number of measurements on environmental indicators that could be acquired by surfers in the UK per month. These statistics were computed as follows. Firstly, the total number of surfs per month were computed for a series of regions around the UK by multiplying Table 1 of Mills and Cummins [49] (the number of surfers per region) with Table 20 of Mills and Cummins [49] (the number of times per month by region that surfers go surfing). The regions defined in Mills and Cummins [49] were then aggregated into six key areas: South-West England = Cornwall + South Devon + North Devon; South coast England = South Coast 1 + South Coast 2 + South Coast 3; East coast England = East Coast + North East; Scotland = East Coast Scotland + Morray Firth + North Coast + Outer Hebrides + Orkney Islands + Inner Hebrides; Northern Ireland = Northern Ireland; and Wales = Cardiff + Swansea + West Wales + North Wales. Total measurements for each region per year are also provided. Note that land locked UK surfers defined by Mills and Cummins [49], who are also estimated to surf ∼ 2.6 × 10^6^ per year, were not included in this analysis as it was difficult to determine surfing locations.

Using a survey of > 2000 respondents, and based on an estimate of 500,000 surfers [48], Mills and Cummins [49] estimated the regional distribution of UK surfers and the monthly frequency of participation. Multiplying the number of surfers per region by the annual frequency of participation gives an estimation of the potential number of measurements that could be collected by surfers per year, assuming all UK surfers were equipped to measure environmental indicators. Based on the study of Mills and Cummins [49], we estimate in the region of 40 million independent measurements on environmental indicators could be collected by surfers in the UK per year. Fig 6 shows the spatial (region) and temporal (monthly) distribution of these estimates. South-West England (Devon and Cornwall) is predicted to have the largest benefit with ∼ 18 million measurements per year, followed by the South coast of England, Wales and the East coast of England with between 5–8 million measurements per year each, and by Scotland and Northern Ireland with ∼ 2 million and ∼ 0.4 million measurements per year, respectively. In all six regions, the distribution of measurements are biased toward autumnal months (when the water temperature is warm and the surf conditions relatively consistent in the UK), with a lower number during winter months (Fig 6). As highlighted by Mills and Cummins [49], their study is not without limitations and these numbers are there to serve as rough estimates. Our estimates of sample coverage based on their study (Fig 6) also assume all UK surfers were to participate in data collection when in reality only a small fraction of the total community is likely to participate in a citizen science project. Nevertheless, even if one in a hundred UK surfers were to collect data it is estimated in the region of 400,000 measurements per year in the UK could be collected by surfers on environmental indicators based on study of Mills and Cummins [49]. This would significantly improve sampling coverage in the coastal zone and impact UK coastal management.

Whereas we have illustrated the potential of environmental data collection by surfers in the coastal zone of the UK (Fig 6), surfing is a world-wide activity and conclusions drawn here resonate globally. Surfers frequent uninhabited (e.g. vulnerable reef environments) and remote regions (some of which are inaccessible by large research vessels), and countries with little or no coastal monitoring and coastal management (e.g. parts of Asia, Africa and South America) that may benefit significantly from such data collection. Furthermore, although we have focused our analysis on data collection by surfers, our results have implications for other recreational marine activities. At the UK coastline (excluding in-land waters) it is estimated that there are approximately: 337,000 kayakers; 362,000 small boat sailors; 190,000 scuba divers; 2.8 million outdoor swimmers; 96,000 water-ski and wake-boarders; 98,000 windsurfers; and 62,000 kite-surfers [48]. Citizen science based projects inclusive of these other recreational activities are likely to improve sampling coverage and benefit coastal management.

## Conclusions

The coastal zone provides huge social and economic benefits to society yet is under threat from an increasing human population and climate change. Coastal management aims to minimise these negative impacts while maximising societal benefits the coastal zone offers. Unfortunately, coastal management is challenged by inadequate sampling of key environmental indicators. With a view to enhance sampling coverage required for better coastal management, we investigated the possibility of using recreational surfers as platforms for monitoring environmental indicators in the coastal zone. To do this, we equipped a recreational surfer with a GPS device and a temperature sensor for a period of one year. The GPS data were used to extract information on surfer performance, whereas the temperature sensor was used to derive estimates of SST, an important environmental indicator in coastal waters.

By comparing the SST data collected by the surfer at Wembury Beach, UK, with data collected from a nearby oceanographic station (L4) and satellite observations, we conclude that recreational surfers are capable of acquiring high-quality data on SST in the coastal environment. Furthermore, useful information on surfing performance statistics can be acquired from the GPS data that may help motivate data collection by surfers. Based on a recent analysis of the UK surfing population, we estimate that UK surfers have the potential to acquire up to 40 million independent measurements on environmental indicators per year around the UK coastline. Such a huge level of data collection is likely to significantly enhance sampling coverage of environmental indicators required to improve and support coastal management. Surfing is a world-wide recreation, and our results have global implications for: coastal monitoring in remote and under-sampled regions; for other marine recreational activities in the coastal zone; and for monitoring other environmental indicators to that of SST, that may be measured by recreational water-users.

## Supporting Information

S1 Data GPS Tracks. Contains 85 GPS tracks (comma separated files) used to estimate surfing performance statistics in the manuscript.
(ZIP)
