# Correction: On the Potential of Surfers to Monitor Environmental Indicators in the Coastal Zone

**DOI:** 10.1371/journal.pone.0162591

**Published:** 2016-09-01

**Authors:** Robert J. W. Brewin, Lee de Mora, Thomas Jackson, Thomas G. Brewin, Jamie Shutler

In Fig 2, the values for the background sea-surface temperature (SST) scale are incorrectly labeled. The label for panel A should be 15.0 16.3 17.7 19.0, the label for panel B should be 16.3 17.0 17.7, and the label for panel C should be 17.4. Please see the corrected Fig 2 here.

**Fig 2:**
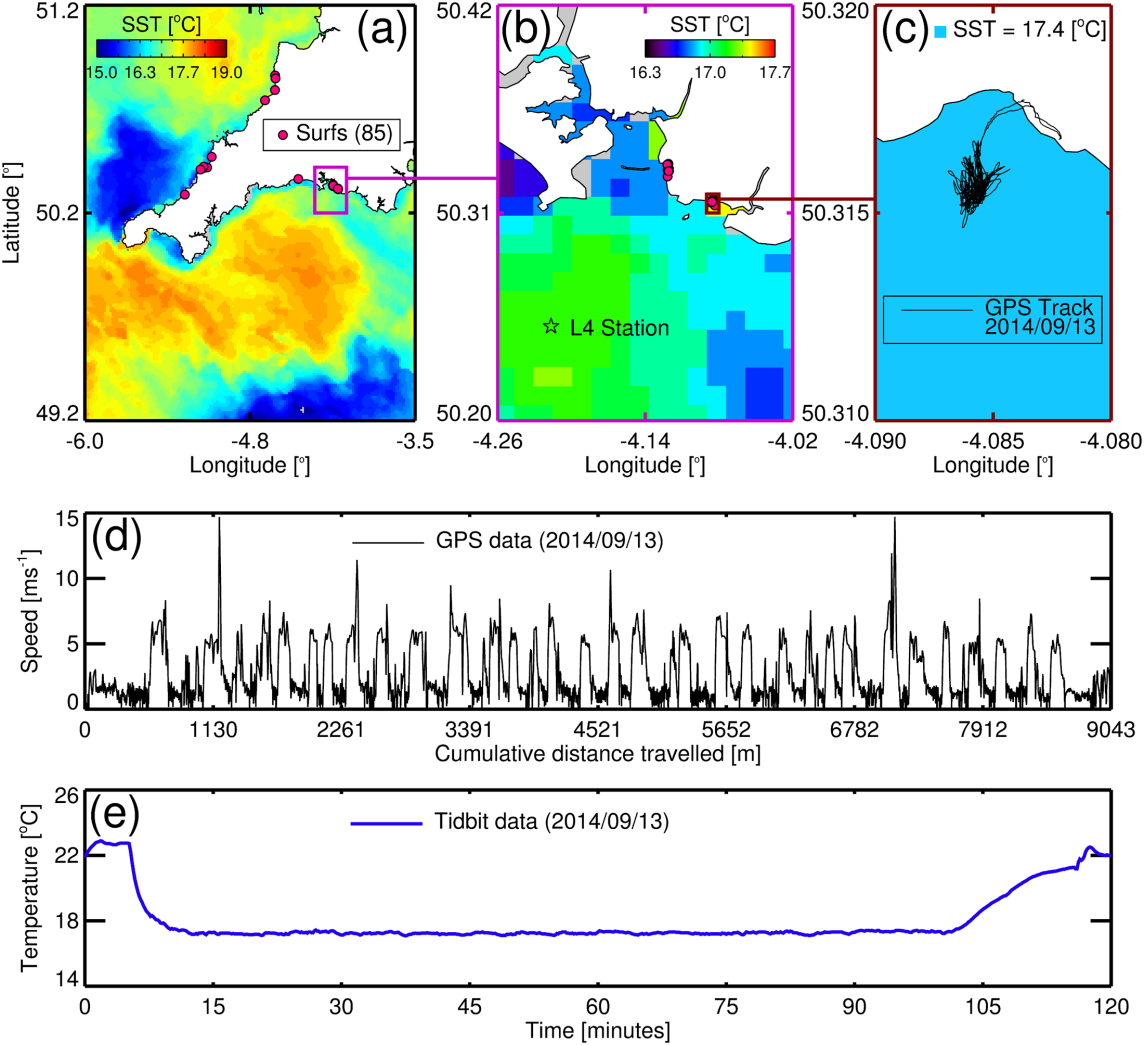
Study site and sampling locations with an example of GPS and temperature data collected by the surfer. (a) Shows the locations of the 85 surfing sessions in South West UK conducted during the study, overlain onto a NEODAAS AVHRR SST image taken on the 10^th^ September 2014. (b) Shows a plot of Plymouth and surrounding waters with locations of the surfing sessions near Plymouth and of station L4 in the Western Channel Observatory, with data from the AVHRR SST image (10^th^ September 2014). (c) Shows a plot of Wembury beach in Plymouth, with a GPS track taken by the surfer on the 13^th^ September 2014 overlain onto AVHRR SST estimate at Wembury beach (10^th^ September 2014). (d) Shows speed as a function of cumulative distance travelled for the GPS track taken on the 13^th^ September 2014, with the bumps in speed indicative of the surfer riding waves. (e) Shows a plot of temperature data collected by the surfer during the surf session on the 13^th^ September 2014.
